# Culturally Diverse Students’ Perspectives on Sexual Violence Policies: Recommendations for Culturally Sensitive Approaches to Prevention in Higher Education

**DOI:** 10.1177/08862605241245372

**Published:** 2024-04-26

**Authors:** Karen Dolores Kennedy, KelleyAnne Malinen, Emily MacLeod, Brooke VanTassel, Kristin O’Rourke, Caryn Small Legs-Nagge

**Affiliations:** 1 Cape Breton University, Sydney, NS, Canada; 2 Mount Saint Vincent University, Halifax, NS, Canada; 3 Halifax Regional Center for Education, Dartmouth, NS, Canada

**Keywords:** sexual assault, cultural contexts, adolescent victims, adult victims, prevention

## Abstract

Culturally sensitive approaches in sexual violence prevention (SVP) refer to the proactive measures and strategies designed to address unique cultural circumstances impacting SVP. It focuses on fostering a culture of consent, respect, and equity and creating a safe and supportive environment for all individuals regardless of your identity. Increasing cultural diversity on university campuses poses unique challenges in preventing sexual violence (SV). Cultural diversity brings different perspectives, norms, and values regarding sex, sexuality, and gender roles. It can contribute to varying understandings of consent, differing attitudes toward SV, and diverse victimization experiences. These differences can create barriers to effectively addressing and preventing SV. The multiphase Culture and Perspectives on Sexual Assault Policy study, conducted at four universities in Eastern Canada, employed a qualitative research design involving focus groups with culturally diverse student participants. The findings revealed a strong desire for more education on sex, sexuality, SVP, and the intersections of culture. Additionally, the findings emphasize the importance of education and comprehensive prevention efforts that consider cultural differences, challenge gender normativity, debunk rape myths, and address the shame and secrecy associated with experiencing SV. These insights have significant implications for promoting a sense of community ownership, increasing the effectiveness and sustainability of prevention efforts, and helping to create a campus environment where all students feel safe, supported, and valued.

This article will address a significant gap in our knowledge and understanding of sexual violence prevention (SVP) by providing culturally diverse students’ perspectives on campus sexual violence (SV) policy, leading to recommendations for culturally based prevention approaches. The findings we present here are a subset of those that resulted from the Culture and Perspectives on Sexual Assault Policy (CAPSAP) study, which aimed to answer the question “How can Nova Scotia build capacity for culturally sensitive sexual violence prevention and response?” As described in the methods section below, we collected perspectives of male- and female-identified students from 14 cultural communities through focus groups that were homogenous for gender and cultural identity. We also held one focus group for students identifying as transgender or nonbinary, which was open to all cultures.

Among our findings, CAPSAP identified the need for proactive rather than reactive approaches in SV policies. Education, a common strategy used in primary prevention (Nation et al., 2003), skills-based training, policy development and enactment, as well as social norms campaigns can provide an upstream approach within the public health framework. Through a public health lens, population-level effects on sexualized violence are most efficiently achieved through a comprehensive, multilevel approach (Dills et al., 2016; Schneider & Hirsch, 2020). Drawing on the input of CAPSAP participants, this article supports pedagogically oriented prevention efforts that address cultural competence in areas such as gender normativity, rape myths, and shame and secrecy. Cultural competence involves incorporating cultural differences to meet diverse needs from a place of awareness that social inequalities interact with cultural differences to shape outcomes (Betancourt, 2003).

SV affects people of all genders, ages, races, and cultures. Despite the fact that most SV is not reported, Burczycka (2019) found 71% of Canadian postsecondary students have experienced or witnessed SV, with an average of 176 per 1,000 students being sexual assault (SA) victims/survivors. SV is a gendered crime in that it is committed primarily against women by men known to them (Boyce, 2013; Mahon, 2016; Ontario Women’s Directorate, 2013), and also in that it is experienced systemically by those who live outside heterosexual norms (Kammer-Kerwick et al., 2021). Moreover, those whose experiences of SV do not fit the man-on-woman paradigm tend to be confronted with social invisibility. According to Statistics Canada (2020) results from the 2018 Survey of Safety in Public and Private Spaces, individuals identifying as Two-Spirit-Lesbian, Gay, Bisexual, Queer, Questioning, Intersex, and Asexual (2SLGBTQQIA+) were nearly three times as likely as their heterosexual counterparts to report incidents of physical or SA within the past 12 months. The study also found that sexual minority Canadians were more than twice as likely as heterosexual Canadians to encounter inappropriate sexual behaviors in public, online, or at work during the previous year.

Kennedy (2022) defines SV as “any sexual act or acts targeting a person’s gender identity or expression, or sexuality against one’s consent, that may occur in person, in writing, by phone, or by any means of communication, including online and social media. SV includes but is not limited to sexual assault (SA), sexual harassment, stalking, indecent exposure, sexual exploitation, and technology-facilitated sexual violence” (p. 5). University-aged women are most at risk of SV, particularly first-year students (Kimble et al., 2008; Senn et al., 2015).

The student population in Canada is increasingly diverse as postsecondary institutions are ever more reliant on international student recruitment (MacLeod et al., 2023). Increased international student enrollment has resulted in a campus environment where intercultural communication skills are crucial. Culture shapes how individuals perceive and process SV (Kalra & Bhugra, 2013). SV is more likely to occur in cultures, including Western society, that foster beliefs in male superiority and female inferiority (Kalra & Bhugra, 2013). Cultural attitudes and gender stereotypes, including an ideology that to be male, one must demonstrate violence and aggression while suppressing vulnerable emotions, contribute to women’s oppression. Hegemonic masculinity crosses all cultures affecting the formation of male–female relationships and the ways in which sexual offenders and victims are viewed (Kalra & Bhugra, 2013). Like gender, race is a socially constructed concept invented and reinvented by people with power to maintain a social order that preserves systems of privilege (Delgado & Stefancic, 2023). Racist and sexist views generate harmful perceptions of women’s sexuality (Harris & Linder, 2017). For example, White women are more likely to be seen as needing protection, whereas Black women are more likely to be seen as mutual combatants (Crenshaw, 1991; Davies, 1981; Duru-Bellat, 2004).

Although interest in and research on SV has gained traction in the past decade, SVP has not been adequately explored from an intersectional approach (Shankar & Tavcer, 2021), which aims to make visible the experiences and needs of marginalized people, including women of color and sexual and gender minorities, whose perspective are not currently adequately represented in the literature (Malinen et al., 2023). According to a study by DeGue et al. (2014), “about two-thirds of the [SVP] interventions reviewed were implemented with majority-White samples” (p. 357). Furthermore, the feminist gender-based approach alone does not adequately consider the complexity of identity. Feminist scholars of color have demonstrated that an exclusive focus on gender when analyzing SV can inadvertently promote a misleading sense of universality that is, in fact, based on the experiences and concerns of white, middle-class, heterosexual women (Crenshaw, 1991; Davies, 1981; Hooks, 1993; Lorde, 1984). Therefore, all the components of oppression, such as gender, race, class, ability, sexuality, nationality, and citizenship (Morris & Bunjun, 2006), must be considered to produce substantive and sustainable change. Although class and ability are identified as factors intersecting with SV, they are beyond the scope of this article and are not addressed. Instead, we are offering suggestions for culturally informed prevention of SV grounded in the data about student cultural diversity collected over the course of the CAPSAP study.

## Background

A search of the literature published between 1980 and 2000 using the terms “sexual violence” and “university” elicits 65 results. The exact same search for 2001 to 2021 provides 4,860 references. This rise suggests dramatically increased acknowledgment of the issue and the need for SVP. Still, universities continue to grapple with developing and implementing robust and inclusive prevention plans and policies. Quinlan et al. (2017) call on university administrators to stop ignoring the problem.

Following a memorandum of agreement between the provincial government and Nova Scotia (NS) universities, the Council of NS University Presidents (2017) published a report with recommendations to address campus SV, SVP working groups were formed, and money was earmarked for SVP research (Province of Nova Scotia & Council of Nova Scotia University Presidents 2017). As a result, NS universities have been working toward SVP and trauma-informed, survivor-centered policy development, meaning policies that focus on the survivors/victims physical, emotional, and psychological safety and well-being, while emphasizing a strength-based approach grounded in an understanding of the pervasiveness of trauma (MacLeod et al., 2023; Malinen et al., 2023). Nonetheless, students continue to study on campuses where many encounter sexism and misogyny (Jackson & Sundaram, 2020).

Globalization adds to the complexity of SVP, necessitating international student input. Canada has seen a 400% increase in international study permits from 2000 to 2021, with upward of 800,000 international students in 2023 (Immigration, Refugees and Citizenship, 2024), underscoring the need for an intersectional approach to SVP. Literature shows that SV, victim blaming, and stigmatization have existed across time and cultures. However, cultural variation exists in how SV is perpetrated, defined, perceived, and responded to (Boy & Kulczycki, 2008; Chan, 2009; Contreras et al., 2010; Damania & Singh, 2022; Dussich, 2001; Equality Now & Dignity Alliance International, 2021; Ilkkaracan, 2015; Ling, 2007; Morley, 2011). In an attempt to support more inclusive SV prevention and response, the CAPSAP project explored culturally diverse student responses to SV policies at four NS universities.

## Literature Review

SV is a population health issue that requires a public health lens and a multidisciplinary approach attending to primary, secondary, and tertiary prevention (Centers for Disease Control and Prevention, 2022), ideally applied at the micro, meso, and macro levels and encompassing culturally competent approaches. A socioecological model interconnects SV interventions at individual, relationship, community, and societal levels (Dahlberg & Krug, 2006). Leveraging the socioecological framework, rooted in Bronfenbrenner’s (1979) ecological systems theory, allows researchers, preventionists, and policy makers to create comprehensive strategies aimed at addressing SV at multiple levels within society. This framework recognizes the complex and layered nature of an individual’s environment, incorporating factors from close groups, broader community contexts, and overarching societal influences. This approach aligns with existing research in the field that also applies a socioecological perspective to understanding SV, as reflected in works by scholars like Victoria Banyard. Banyard’s work consistently applies a socioecological lens to studying violence prevention, particularly in the context of bystander intervention and community-based strategies to prevent SV (Banyard et al., 2009; Moynihan et al., 2011). Schneider and Hirsch (2020) have suggested that effectiveness of SVP strategies would be enhanced by comprehensive sexuality education beginning in early school years and continuing into adulthood.

DeGue et al.’s (2014) systematic review determined that the large majority of SVP prevention studies did not meet the standard for successful prevention as set by Nations et al. (2003) requiring (a) comprehensiveness, (b) diverse instructional methods, (c) adequate dose, (d) theory-based, (e) nurtures relationship building, (f) properly scheduled, and (g) sociocultural. However, Coaching Boys into Men (Miller et al., 2012) and Bringing in the Bystander (Banyard et al., 2009) were identified as having substantial potential for impacting sexually violent behavior.

More recently, Mujal et al.’s (2021) systematic review of SV bystander training concluded that numerous bystander programs had been found to decrease rape myth acceptance (Amar et al., 2012; Banyard et al., 2009; Moynihan et al., 2010), increase bystanders’ confidence and willingness to help (Amar et al., 2012; Banyard et al., 2009; Moynihan et al., 2010, 2011; Senn & Forrest, 2016), and to a lesser extent positively impact attitudes and behaviors (Jouriles et al., 2016; S. McMahon et al., 2015; Moynihan et al., 2015; Senn & Forrest, 2016). Unfortunately, bystander programs have been studied with majority white respondents and mostly without pre-test/post-test evaluation (Mujal et al., 2021). Despite the many benefits of bystander training, SV often occurs in a private environment where ally intervention is not possible, pointing to the need for the addition of individual perpetrator behavior change. Changes in perpetrator behavior require changes to the status quo or unlearning and relearning social norms.

Perceptions of social norms, extrinsically developed attitudes and behaviors, significantly impact individual behavior, which may or may not align with individual attitudes and behaviors (Berkowitz, 2010; Bingenheimer, 2019). Arguing for the importance of education, Schneider and Hirsch (2020) note that SVP, which has to date been largely ineffective, would benefit from comprehensive sexuality education beginning in the early school years.

A change in social norms can be achieved through (a) a comparison of one’s behavior to that of others, (b) marketing campaigns suggestive of others’ positive behavior, and (c) small group discussions that explore old behaviors and the need for new behaviors (Cislaghi & Heise, 2020). Changes in social and gender norms require updates and improvements in organizational policies, individual narratives, power dynamics, and media outputs (Cislaghi & Heise, 2020). A complex problem such as SVP also requires a comprehensive approach that targets perpetrator behavior (Schneider & Hirsch, 2020), and programs that have been evaluated as effective (DeGue et al., 2014). We suggest that SVP in universities include a culturally competent approach to constructs such as shame and secrecy, gender normativity, and rape myths.

A variety of interrelated approaches have been advanced as methods for enabling positive intercultural interactions in educational or caring contexts. These include “Cultural Literacy” (Wood et al., 2006), “Cultural Safety” (Brown et al., 2016), and “Cultural Humility” (Foronda et al., 2015; Tervalon & Murray-Garcia, 1998), which focus on various modalities of attentiveness to one’s own culture in relation to openness to and awareness of diverse other cultures. “Cultural competence,” the framework taken up here, is among frameworks that go beyond reflexivity, openness, and general skills for intercultural communication to advocate acquiring knowledge about characteristics of the cultures present in a given interaction. As Ono (2013) suggests, a risk of approaches to intercultural communication that rely on acquiring specific knowledge about exogenous cultural communities is that ideological assumptions may form the foundation for cultural comparison. However, many of our participants emphasized that it is essential for university representatives administering SV policies and services to understand how SV is seen and responded to in their home countries and communities. Thus, we have taken up the framework of cultural responsiveness while cautioning that it is essential to be aware of vast diversity within cultural communities, as well as the fact that many people live at the intersection of several cultures.

In addition to cultural competence, using an intersectional lens helps us understand why individuals with a combination of marginalized identities are at greater risk of experiencing SV. The term intersectionality was developed by Crenshaw (1991), an American law scholar and civil rights activist, to describe how race and gender interact to create obstacles for Black women. Intersectionality is grounded in Black feminist theory and is inspired by the activist movements of women of color in the 1960s and 1970s. The phrase first drew attention to anti-discrimination legislation that did not adequately safeguard the rights of Black women (Crenshaw, 1991). More specifically, intersectionality emerged to make marginalized women’s unique experiences more visible (Crenshaw, 1991). Since its inception, intersectionality and intersectional feminism have evolved and expanded beyond race and gender to include “the interaction [among] categories of difference in individual lives, social practices, institutional arrangements, and cultural ideologies and the outcomes of these interactions in terms of power” (Davis, 2008, p. 68). In intersectional approaches, there is an emphasis on how social categories, such as race, class, ability, gender, and sexuality, interact to shape experiences of SV (Crenshaw, 1991). As a result, individuals who have experienced SV and who are contending with multiple oppressions may encounter challenges that are not adequately addressed by care practices (Kulkarni, 2018). Furthermore, intersectionality recognizes collective traumas resulting from historical trauma, institutional racism, poverty, and colonialism. Considering SV with an intersectional lens requires scholars, activists, and practitioners pay close attention to the nuance and context of an individual’s existence and their subsequent experiences (Gill, 2018).

As part of intersectionality and cultural competency as they relate to SV, sociocultural contexts of shame and secrecy, which were commonly discussed by CAPSAP participants, require consideration. Ferreira et al. (2022) define shame as “. . . a particularly intense and often incapacitating, unwanted emotion involving feelings of inferiority, defectiveness, powerlessness, uselessness, isolation and self-consciousness, along with a desire to escape, hide or conceal deficiencies” (p. 1832). Feelings of shame can be categorized as internal (judging yourself) or external (being judged, or a perception of being judged by others), resulting in personal devaluation. Internal shame and external shame are closely linked, exacerbating one another, which ultimately contributes to victim blaming (Cherniawsky & Morrison, 2022). Studies have shown that law enforcement officers often subscribe to rape myths associated with false reporting, and this alignment has implications for their decision-making processes and the advancement of SA cases (Dewald & Lorenz, 2022). Survivor/victims who report to police and have evidence collection obtained often feel betrayed when evidence is not tested, furthering victim blaming by family and friends (Campbell et al., 2024). Patriarchal societies enforce sociocultural norms such as shame, which may serve as a political tool to silence women, reinforcing male domination. Social beliefs related to honor, femininity, and masculinity can result in survivors/victims being held more liable than the offender for dishonoring the family and community (Zakarriya, 2019). In this way, shame as it relates to SV is deeply related to gender norms.

Gender normativity is intertwined with heteronormativity, which enforces the idea that only intimacy between men and women is right or normal. Feminist scholars argue that heterosexual and even same-sex relationships take form in a “rape culture” in which SV is normalized (Malinen, 2018; Pascoe & Hollander, 2016). Rape culture is also seen to play out in SV that occurs within queer communities (Malinen et al., 2013). SV, SA, and threats of such are deeply integrated into the fabric of young people’s lives such that they become a normative instead of an unusual aspect of their emotional and physical relationships (Pascoe & Hollander, 2016). Rape culture stems from historical gender norms for what it means to be either female or male.

Gender stereotypes encourage men to display dominance over women and other men, while historically, in many cultures, females are thought to be submissive to men. The concept of rape myths was first introduced by Burt (1980), who defined them as “prejudicial, stereotyped, or false beliefs about rape, rape victims and rapists” which created a “climate hostile to rape victims.” The concept was further explained by Lonsway and Fitzgerald (1994), who added that rape myths are best understood as stereotypes, explaining that they are “attitudes and beliefs that are generally false but are persistently held, and that serve to deny and justify male sexual aggression against women” (p. 134). Rape myths can be defined as inaccurate cultural beliefs blaming victims for the SA and denying or justifying violence against women (Hockett et al., 2016), and have been found to negatively impact a bystander’s response to witnessed SV (Banyard & Moynihan, 2011).

Most rape myths are directed at the survivor of the violence, focusing on their behavior or demeanor as the cause of what happened, limiting who can be viewed as a credible victim. Further, rape myths differ by culture (Damania & Singh, 2022) and gender. For example, males who report their SV victimization must not only deal with similar issues that females face when reporting, such as disbelief but also must contend with structural barriers due to lack of services and support (O’Connor, 2021; Walfield, 2021). Universities and stakeholders must recognize various types of systemic oppression and those factors contributing to instances of SV among university populations. Changing norms can advance prevention by deconstructing rape myths can help to decrease the endorsement of distortions used to justify SV and to dispel various gender stereotypes (Walfield, 2021).

## Methods

The CAPSAP project was a multiphase, multisite, qualitative study that took place at four postsecondary institutions across NS, Canada. The authors of this paper are co-researchers of the CAPSAP study and have research interest and practice expertise in community and campus SV education, prevention, and response. Their combined experience enabled the recruitment, training, and development of trust with student research assistants (RAs) who facilitated focus groups. Participants and RAs comprising each focus group shared the same self-identified gender and cultural identity. RAs received an 11-hour training that introduced the CAPSAP study and covered all aspects of fieldwork for which RAs were responsible (recruiting, conducting, and transcribing focus groups), including ethical considerations and trauma-informed approaches.

Each participating institution had an SV policy that included processes for reporting and investigation of SV on campus as well as avenues for accessing supports. Focus groups began with a facilitator presentation about their university SV policy, lasting approximately 15–20 minutes. Following these presentations, student participants were provided the opportunity to offer their perspectives on campus SV policies and services during semi-structured focus group discussions. The purpose of the study was to answer the question, “How can Nova Scotia universities build capacity for culturally sensitive sexual violence prevention and response?” In seeking responses to this question, we chose to speak to culturally diverse students rather than campus-based service providers because we believed students would have culturally specific insights into their own concerns, questions, needs, and experiences with campus life in NS. Discussions included participant perspectives on forms and qualities of campus-based SV prevention and response that might be helpful or harmful for students belonging to their cultural communities. This article represents a subset of the findings generated by our study.

### Sample

In phases one and two of the CAPSAP study, 42 focus groups, with most groups hosting 5 to 7 participants, were held at four universities in Atlantic Canada. Represented communities included African Nova Scotian; Canadian; European; North African, North Indian, South Asian, South Indian, West African, Caribbean, Bermudian, Bahamian; Chinese; East African; Latin American; Southeast Asian; Turkish; and Middle Eastern. Participants in each focus group, including student RAs, were matched for gender identity. Male and female-identified groups were matched for cultural identity, whereas one focus group comprised of transgender and nonbinary participants was open to all cultural identities. Same gender groups were deployed because people are often more comfortable expressing thoughts and feelings about SV in same gender groups, and because of the interconnection of gender with prevalence, experiences, visibility, and social reception of SV (Boyce, 2013; Burczycka, 2019; Kennedy, 2022; Kimble et al., 2008; Mahon, 2016; Malinen et al., 2023; Ontario Women’s Directorate, 2013; Senn et al., 2015). Similarly, culturally homogenous groups were intended to enhance comfort and mutual understanding, and to support discussion about cultural experiences in Canada and “at home” among in-group members.

### Recruitment

Student focus group participants were recruited purposively and using a snowball approach. Initial student participants were identified using the student RAs’ personal networks. Student participants then identified additional potential participants from within their social networks. Although selection bias is a risk of using a snowball method and social networking for recruitment (Sadler et al., 2010), the breadth and extensiveness of the study in terms of participating institutions and the number of focus groups significantly reduce this limitation.

### Data Collection and Analysis

Phase one of the study consisted of face-to-face focus groups at one university, whereas, in response to the COVID-19 pandemic, phase two focus groups were conducted with students from three other universities in virtual meeting spaces by student RAs. Student participants reviewed their respective institutional SV policies and were led through a discussion based on a semi-structured discussion guide with follow-up prompts. A total of seven questions were designed to evoke reflection and open-ended conversation among participants. One example is, “How do you think older members of your family would respond to this policy, whether parents, aunts, uncles, or grandparents? Tell me who you are thinking of and what would they say?” This question evoked a great deal of conversation about the changes participants saw occurring in their own cultures over time, the role of their upbringings in how they thought about SV, and the availability or unavailability of family supports in the case of SV. Other questions included “What kind of characteristics or qualities do you think a person needs to have to support someone who has experienced sexual assault?” and “If you could offer advice to folks at our university, like security staff or counsellors, who might support people from [your cultural region] who have experienced sexual assault, what advice would you offer?” These aspects of focus group discussions generated participant ideas about how survivors should be supported in general as well as suggestions of things service providers should but might not know about the cultural backgrounds of their students. Follow-up prompts were used by facilitators to delve deeper into topical threads as they arose.

Focus group discussions were audio recorded and transcribed by student RAs. On average, discussions lasted approximately 60 minutes. All student participants provided written and oral consent. The CAPSAP study received ethics approval from the Research Ethics Boards at all four participating universities. Despite our inability to recruit Indigenous participants, the CAPSAP project did receive ethics approval from Mi’kmaw Ethics Watch. Transcripts were read and reread for verification.

Through examining the data, members of the research team identified a wide range of perspectives held by students of diverse genders and cultures. Transcripts from each university were coded using a constant comparative method within and sequentially across interviews (Glaser et al., 1968). Discrepancies in coding were resolved through discussion and consensus among co-researchers. During the coding process, a public health framework was applied by members of the research team as qualitative data aligned with primary, secondary, and tertiary prevention themes were identified. Broad themes from the analysis were presented to the focus group facilitators and end-users (e.g., members of university student affairs, international student center representatives, coordinators of the provincial Sexual Assault Nurse Examiner [SANE] program, and content experts in the field of women and gender studies). Analyses of the data and review of current literature resulted in publicly available reports specific to each participating institution. The reports listed recommendations for the amendment and implementation of policies and strategies, and initiatives to rectify identified gaps, thus increasing student safety.

## Results

Feedback from students belonging to various cultural and gender groups revealed that they want and feel they need more education related to sex, sexuality, SVP, and the intersections of culture. In the words of a West African Female participant, “This policy is more [for] afterwards. [. . .] What are the preventative measures this school can provide? [. . .] How can you prevent this from happening to you?” Additionally, Centers for Disease Control and Prevention (2017) recommends using a socioecological model for SV prevention. This model provides a multipronged approach to prevention, allowing for interventions at the individual, relationship, and community levels (Basile et al., 2016). For the purposes of this article, we will explore four subthemes that emerged under the higher-level theme of education: cultural differences, gender normativity, rape myths, and shame and secrecy.

### Gender Normativity

Student participants from various cultural backgrounds recognized that people of any gender or sexual orientation can experience SV. Male participants expressed concerns about being marginalized and silenced, noting that their reality is often not represented in SV policies. Furthermore, some male focus groups identified a common misconception that men cannot be sexually violated.

North African Male: *No matter your background, 90% of the time it’s assumed that the female has more power to go and say, “I’ve been sexually assaulted,” and they will have more help. I think it’s time to say, no matter if you are straight, or gay, or male, no matter your identity or gender, you could have faced sexual violence. Especially for straight men, people always assume that straight men will not face sexual violence.*

African NS Male: *If the example is “A girl got me drunk one night and took advantage of me, or sexually assaulted me,” for me to come home and tell my teammates that, they might be like, “Shit, what are you complaining about? What’s the issue?” And again, that’s a whole different toxic masculinity issue. But it’s the reality in our lives as Black men*.

The final sentence of the second passage above exemplifies how many participants brought intersectional lenses to their analyses, thinking simultaneously in terms of race or ethnicity and gender.

Participants also discussed the socialization of men, wherein aggressive and dominant behaviors are expected and contribute to men most often being the perpetrators of SV. Consistent with the approach taken by Katz (2018) and his trailblazing Mentors in Violence Prevention program, participants felt that solutions to SV must include and address gender norms and stereotypes. The Chinese Female participant cited next took an intersectional perspective in considering systems that shape individual experiences of SV:

Chinese Female: *. . . This University [. . .] should understand there is a fact that each person’s experience will be affected by many factors including, you know, their sex, their—their race, language, ability, age, or even gender identity. . . . Sexual violence can also be acts of, like, the systematic oppression including sexism, racism, sometimes the homophobia or transphobia. So, I think, uh, the policy needs to include them all, or need to take all the facts into considerations. Yeah, thank you*.

### Rape Myths

Numerous focus groups across cultures and genders discussed rape myths directly and indirectly. For example, some cultural and gender groups spoke of the need to denounce current rape myths related to how one is dressed, who can be assaulted, and where an assault occurs. Other focus group participants unknowingly perpetuated rape myths by making statements about the need to stay away from dangerous places and strangers, dress appropriately, and be careful of others from different cultural and social backgrounds. Many student participants also espoused self-blaming attitudes. Additionally, many male participants appeared deeply concerned about wrongful allegations of SA, a fear-driven at least partly by the myth that men are often accused falsely. Rape myths were not noted within the transgender and nonbinary group. Because only one group was held for this gender identity, it cannot be concluded if this is reflective of a trend among transgender and nonbinary people. Generational transformations in understanding rape myths were suggested by many participant discussions in that participants disagreed with victim blaming and shaming beliefs held by their parents and grandparents.

One West African Female participant expressed their view of what counted as SA, based on the view that a woman consents to sexual activity with a man merely by virtue of being alone with him.

West Africa Female: *Okay, so basically, sexual assault for me is –* [. . .] I don’t know how *to put it. So, basically, if there is a guy, and let’s say [. . .] I am not ready to do anything sexual with the guy. I would never put myself in a position where something like that would happen. If I am going to be in a enclosed space with the guy, I will be ready for anything to happen. Do you understand? So, sexual assault for me would only be if I did not put myself in that enclosed environment. Maybe I was dragged [laughter] to an enclosed environment and then drugged. To me, that is sexual assault.*

Other West African participants disagreed that being alone with a man constitutes consent. Canadian participants also identified victim blaming as part of their culture.

Canadian Female: *I think with both my mom and dad there is a disconnect in their understanding of sexual assault. You know, when there is cases and stuff like that on TV, you know, they say stuff like, “Oh, she’s fibbing,” and “She needs to get over it,” and putting the blame back on females. So, I think that there would be some conflicting feelings about this [the policy] because they would feel that, um, that people would be more apt to cry wolf.*

Chinese participants in male and female focus groups alike articulated a belief that Chinese people should protect themselves by staying away from a range of places and people, and by not going out alone.

Chinese Male: *I think. . .people in China should keep [away from] some dangerous places, like night clubs, as they protect themselves; and they should know. . .hmm, where—where, what kind of people are, and keep themselves from those, er, remote places.*

Chinese Female: *It’s not good to be alone when you are in public. [I] feel it’s easy to get trapped if you got seduced by someone from a different background, different religion, different countries. So, uh, my parents will also, uhm, tell me to be alert and don’t be alone in public as much as you can.*

### Cultural Differences

SV was perceived differently across cultural groups. Many groups expressed the need for education on Canadian law related to SV. Student participants also shared the desire for intercultural educational opportunities to understand and respect each other’s differences. Some participants spoke about the meaning of sex, sexuality, and, consequently, what defines SA in their culture. Some participants believed that what one person perceived as being friendly and inviting might be considered a threat by another, depending on culture, perhaps resulting in SV allegations based on an honest misunderstanding. Students often suggested that their ideas about SV were instilled in them by parental figures or societal norms.

North India Male: *Some people might be thinking that that is being friendly. Some people might be thinking that, you know, that, this is how, like, things have always been where I’ve grown up. But things are different here, and I find that a lot of people get into trouble because they don’t know that things are different.*

Caribbean Female: *The relationship dynamics within the Caribbean, like, every relationship is different, but a lot of behaviours are normalized in our culture that would be seen as—I don’t wanna say abuse—like, basically, a lot of sexual violence comes from relationships. I mean, a lot of times they are people that you interact with. It’s just, like, just cultural dynamics, friend dynamics, and those kinds of things from not only our culture, but all the different cultures present in the university environment.*

The preceding transcript passages point to distinct cultural differences in norms for dating and intimate relationships, highlighting the need for unique culturally specific SVP solutions. Another consideration is expressed by a West African participant who suggested the experience of SV transcends culture.

West African Female: *But. . . it’s—every women feels the same. We may physically have different cultural backgrounds but when it comes to sexual harassment, we have—we feel the same. It doesn’t matter where you are from.*

### Shame and Secrecy

Students from all institutions and various cultures expressed concern for their personal and familial reputation if they experienced SV. The shame/stigma surrounding SV fueled fears of isolation and concerns about negative impacts on the potential for future relationships and marriages. Given the social repercussions, participants shared their hesitancy to report. They described a culture of conservatism as prohibiting survivor speech. Although some male-identified participants shared worries about the shame associated with wrongful accusations, no participants (male or female) pondered the shame perpetrators might bring to themselves, their families and their communities. Instead, shame seemed to be reserved for survivors/victims. The impact of shame and secrecy brought forth concerns about confidentiality and anonymity. More specifically, students spoke of their hesitancy to report SV out of fear that their identity would be exposed—tarnishing their reputations and their families’ reputations.

Chinese Female: *For example, I am from China, I am pretty much a very traditional and conserv[ative] person. [. . .] Most Chinese girls are pretty much traditional. Like, your shame to talk [about] all this—the—the sexual things in public, right?*

Chinese Female: *First of all, we all know that this is something that can damage the honor of that friend. What if her future boyfriend knows about this? What if her future husband know this? Right? You need to understand what she thinks. It could even [. . .] leave her with PTSD afterwards. Some students, especially international students here, who are from places where sex is a taboo topic, or even you get a penalised for adultery*.

## Discussion and Recommendations

With the application of a public health lens, culturally competent prevention efforts emerged as a prominent theme for participants from all cultural groups. Arising from a public health framework, primary prevention, which includes efforts or strategies to intervene before an assault occurs, is arguably the most critical component of SVP (DeGue et al., 2012; Wells et al., 2012). Such strategies focus on perpetrator prevention instead of placing the onus on potential victims/survivors. Our findings suggest education-based prevention efforts that are informed by the culturally diverse perspectives and experiences of campus community members would be useful. More specifically, it would be useful to (a) promote forms of masculinity that are not consistent with perpetrating SV; (b) emphasize that one’s clothing, willingness to communicate with strangers, location, or the time of day or night never constitute consent; and (c) ensure students know what constitutes SV in Canada. Interventions in these areas would help to alleviate the climate of shame and secrecy by removing the onus of prevention and blame for SA from the survivor/victim.

Perceptions of SV varied among cultural groups, with many expressing a need for education on Canadian laws related to SV. Student participants also conveyed a desire for intercultural educational opportunities to foster understanding and respect for differences. Discussions delved into the cultural interpretations of sex, sexuality, and the varying definitions of SA. Some participants highlighted the subjective nature of interactions, noting that what may be perceived as friendly by one person could be seen as a threat by another, leading to potential misunderstandings and allegations based on genuine misinterpretations. Students frequently indicated that their perspectives on SV were shaped by parental figures and societal norms.

Institutions must consider accessibility and cultural competence as they plan SVP education (Rawson & Liamputtong, 2010; Usher et al., 2017). Students from many focus groups identified this concern, but it was predominantly evident in students from Southeast Asia and China. Such culturally informed education strategies may be targeted at the individual, community, or institutional level. Some researchers criticize individual approaches focused on prevention tips (e.g., such as self-defense classes, or clothing choices), attributing this type of prevention to victim blaming (Cherniawsky & Morrison, 2022; Rentschler, 2015). Individual strategies by themselves may not significantly impact SVP; rather, they are one cog in the wheel of SVP. Individual approaches to SV are best incorporated with meso- and macro-level approaches. For example, primary prevention at an institutional level might include peer-led group classes focusing on topics such as sexuality, healthy relationships, anti-racism, and challenging gender normativity.

We suggest the following strategies to enhance intercultural education with the aim of SVP in postsecondary institutions:

- *Diverse peer support networks*: Create an interuniversity and college roster of culturally diverse peer supporters and educators, including international and domestic student leaders across genders.
- *Development of cultural responsiveness*: Engage in activities to foster cultural responsiveness specific to SVP and response, applying culturally competent practices to ensure culturally appropriate prevention efforts are inclusive and respectful.
- *Inclusive education*: Provide culturally competent education to all incoming students about what constitutes SV in the Canadian context.

*Anti-racist education*: Develop and implement anti-racist education and training for service providers and investigators involved in SV cases. Our analysis suggested the importance of education in the areas of shame and secrecy, cultural differences, gender normativity, and rape myths. Students from various cultural groups identified a need for education on SVP, due to ongoing prevalence rape myths and gender normativity. Peer-led interpersonal education was identified as a possible method for improved cultural understanding related to SV. Students felt that it was necessary to not only know the laws and culture of NS, but also to provide a safe space where students can discuss differences among diverse cultures within the university. Giving students the opportunity to discuss issues such as SV and gender normativity in a safe, intercultural environment will allow the exploration of diverse ideologies, those that have emerged from a western context, and how they contribute to instances of SV. Additionally, providing a safe space for information sharing might increase respect for diverse cultural boundaries and norms and decrease the shame and secrecy associated with topics of sex, sexuality, and SV.

SV is a challenging topic for many university students to discuss. One reason survivors/victims do not report SV is the shame associated with sexual violation (Swartz, 2018). This finding was reaffirmed in the CAPSAP study. In many countries, conversations about sex, sexuality, and SV are unwelcome. Students from many focus groups spoke of the need for more knowledge regarding SV, SVP, and SV policies before arriving in NS and during their postsecondary education. Similar results were found in a study conducted by Magnussen and Shankar (2019), where some students did not know that an SV policy existed, whereas others noted that they did not know where or how to access it. It was clear from participants across various cultures and genders that institutions are inadequately disseminating SVP policies and reporting pathways (Magnussen & Shankar, 2019).

Focus groups felt that inadequate knowledge and limited awareness of SV and SV policies arose from poor institutional dissemination intersecting with dynamics of shame and secrecy in various cultural communities. Furthermore, they spoke about many cultures in which it is not normative to engage in conversations about healthy sexual relations, perpetuating bidirectional of shame and secrecy associated with SV. Silence about SV and the propensity of societies to victim blame may originate from societal and cultural norms, which are maintained by the silence they produce (Fessler, 2007).

Sociocultural norms impact how survivors/victims process SV. For example, shame, honor, and patriarchal structures in Southeast Asian communities influence SV reporting and disclosure (Cowburn et al., 2015). During the focus groups, many Southeast Asian participants spoke of shame and dishonor, indicating that the fear of being exposed as a victim of SV is a barrier to reporting and help-seeking. Survivors/victims often considered reporting SV to be risky due to the potential for negative family and community reactions. Nonetheless, participants from these communities felt that those who speak of their assaults are brave and deserving of recognition. Contrastingly, some focus group members shared the need to report SV as it is essential for improved well-being. Regardless of its origin, shame and secrecy impact preventative and restorative health measures, highlighting the importance of anonymous avenues for SV disclosure and support. Regrettably, there are missed opportunities for accommodation, treatment, and support each time students choose not to share information about their assault. The fear and experience of shame halts survivor speech and removes access to services essential to student health and well-being (Sutherland et al., 2016). More directly linked to the topic of this article, a climate of shame, secrecy, and underreporting is a climate in which potential perpetrators may feel it is safe to commit SV.

We offer a number of recommendations to address the shame and secrecy associated with SV within higher education.

- *Visible support structures*: Enhance accessibility to on- and off-campus support and policy information by strategically placing QR codes in high-traffic areas. Ensure that all materials related to SVP are available in multiple languages and use culturally competent language to avoid reinforcing stereotypes or perpetuating biases.
- *Anonymous inquiry platforms*: Implement an anonymous app or tool enabling students to ask questions about SV and university policies, ensuring confidentiality, safety, and cultural competence in prevention efforts. Consider diverse communication styles and preferences.
- *Policy awareness campaigns*: Develop and promote awareness campaigns that specifically address cultural aspects of SV, challenging stereotypes, and promoting respectful behavior. Utilize various communication channels and platforms to reach a diverse audience.
- *Promotion of open dialogue*: Provide time and spaces for intercultural peer-led discussions about sex, sexuality, and healthy relationships, integrating cultural safety considerations to create an inclusive environment that supports prevention efforts.

Our analysis of participant discussion indicated that university programs and policies may impact SV through the incorporation of a culturally informed prevention plan that attempts to interrogate relevant assumptions about gender roles and encourages change in gendered expectations that support SV. SV education, programming, and policies might benefit from a focus on understanding rape and SV in the context of gender inequalities and norms. As suggested by Katz (2018) productively reorienting gender norms could include socialization of young men to use their masculinity for good, for example, by asserting themselves to intervene as effective bystanders. For these reasons, we offer the following recommendations:

- *Culturally diverse masculinity campaigns*: Conduct education and awareness campaigns promoting culturally diverse, nonviolent forms of masculinity.
- *Inclusive discussions*: Include male students from diverse backgrounds in discussions of SV, gender stereotypes, and stereotypes of masculinity.

According to Hockett et al. (2016), rape myths are prejudicial, stereotyped false beliefs about rape, survivors/victims, and rapists. A plethora of rape myths exist, some of which were espoused by study participants. For example, some study participants espoused the notion that perpetrators are mainly strangers; that males cannot be victims of SV; or that a survivor/victim acts or dresses in a particular way, enticing their offender. Hockett et al. (2016) have suggested that rape myths reinforce a societal hierarchy of male dominance and that rape myths are designed to intimidate women into submission. Relatedly, many focus groups talked about the need to educate university students in an effort to raise awareness of gender oppression. Some student participants spoke about rape myths creating a culture wherein many survivors/victims feel blamed for their victimization and, therefore, hesitant to disclose (Caron & Mitchell, 2022). Another misconception identified by participants across many cultural groups is an understanding of SV that excludes the possibility of male victimization, which ought to be challenged in the interest of supporting all survivors/victims.

Crocker et al. (2020) assert that about two-thirds of the interventions within institutional SV policies include educating and training the campus community about the need to challenge rape myths, with a specific emphasis on confronting victim blaming. Additionally, our research identified the need for interventions and policies that include a cultural competence lens. The importance of such measures was also emphasized in many focus groups. On the one hand, ideologies that perpetuate the narrative that women need to be cautious in their actions or should not be alone in public were espoused in some focus groups. On the other hand, study participants spoke about rape myths and the need to reverse common misconceptions about SV. Reductive ideologies about who can be violated, who violates, where and how, what constitutes SV, and rates of false accusations were identified by some participants and by the researchers as rape myths that must continue to be deconstructed through diverse educational strategies.

Power, privilege, and oppression in various forms must be understood as factors shaping SV; failing to broaden the conversation in these areas will curtail eradication of SV (Harris & Linder, 2017). Students who experience multiple oppressions or marginalization because of their intersecting identities, such racialized or ethnic minorities, often perceive and experience available resources and campus processes as risk-laden while being more vulnerable to SV (Harris & Linder, 2017).

Acknowledgment of these issues is imperative if university institutions are to provide education to students and faculty that deconstructs ideologies impacting marginalized groups. Faculty could spearhead education opportunities to combat SV, focusing on the intersections of students’ identities so that all institutional members gain knowledge and understanding of the experiences and histories of students of diverse and marginalized communities. Courses and educational material that address race, gender, and the intersections of identity will challenge dominant ideologies by focusing on topics that center marginalized groups as those disproportionately affected by SV (Harris & Linder, 2017). By understanding the needs of racialized groups on campus, administrators and faculty are better equipped to form policies that address all campus communities and critique interlocking systems of oppression (Harris & Linder, 2017).

In keeping with the feedback received from study participants, addressing the intersectional needs of campus community members may involve the following culturally competent approaches.

- *Myth deconstruction*: Ensure all SVP and response education and training opportunities actively deconstruct victim blaming, rape myths, and gender norms.
- Challenge stereotypes by highlighting that a significant percentage of men from various cultures have experienced SV in their lives.
- *Intersectional approach*: Ensure educational sessions take an intersectional approach to understanding SV and supporting victim/survivors.
- *Legal education with cultural competence*: Deliver accessible education about Canadian SA laws, highlighting their practical implications and challenges faced by survivors.
- *Consent education with cultural awareness*: Create and promote avenues for comprehensive consent education, describing legal and institutional consequences for respondents who have engaged in SV, while indicating the range of consequences likely to follow various policyviolations.

## Conclusion

Lumby (2012) asserts that a deep understanding of culture is required for successful cultural change; if culture is addressed superficially, institutions will likely perpetuate the status quo (Ahmed, 2012). Given the large number of international students in Canadian universities, it is critical to understand their perspectives on SV policies, and on this basis, to provide university administrators, student services personnel, and policy writers with culturally relevant recommendations for inclusive SV policies. The CAPSAP study provided essential data to inform key recommendations for culturally competent primary prevention strategies impacting SV. More specifically, this article explored transcript passages related to gender normativity, rape myths, shame and secrecy, and cultural differences, providing context for the associated recommendations. We hope that this research will contribute to a broader intersectional project of addressing institutional and cultural barriers that may currently prevent many members of our campus communities from engaging with SVP initiatives. Broadly speaking, we recommend that postsecondary institutions and governments invest in research and evaluation focusing on the impact of culturally responsive SVP initiatives.
